# Endoscopic ligation of the anterior ethmoidal artery: a cadaver dissection study

**DOI:** 10.1590/S1808-86942011000100006

**Published:** 2015-10-19

**Authors:** Bernardo Cunha Araujo Filho, Carlos Diógenes Pinheiro-Neto, Henrique Faria Ramos, Richard Louis Voegels, Luiz Ubirajara Sennes

**Affiliations:** 1 Doctoral degree in otorhinolaryngology, Medical School, São Paulo University. Otorhinolaryngologist, residency at the Clinical Hospital, Medical School, Sao Paulo University (HCFMUSP). ENT specialist (ABORL-CCF); 2 Doctoral student in otorhinolaryngology, Medical School, São Paulo University. Clinical Instructor of the Department of Otolaryngology, University of Pittsburgh School of Medicine; 3 Doctoral student in otorhinolaryngology, Medical School, São Paulo University. Physician of the Otorhinolaryngology Clinic, IAMSPE; 4 Associate professor of the Clinical Otorhinolaryngology Division, São Paulo University. Director of rhinology of the Clinical Otorhinolaryngology Division, Clinical Hospital, São Paulo University; 5 Associate professor of the Clinical Otorhinolaryngology Division, São Paulo University. Coordinator of the graduation course in otorhinolaryngology, Medical School, São Paulo University

**Keywords:** ethmoidal anterior artery, endoscopic, endoscope, epistaxis

## Abstract

Anterior ethmoidal artery (AEA) ligation may be necessary in cases of severe epistaxis not controllable with traditional therapy. Endoscopic endonasal ligation of the AEA is not used frequently; there are few studies in the literature for standardization of the endoscopic technique for this vessel.

**Aim:** To demonstrate the feasibility of periorbital AEA ligation in a transethmoidal endoscopic approach.

**Methods:** A prospective study where 50 nasal cavities were dissected. After anterior ethmoidectomy and partial removal of lamina papyracea, the periorbital area was carefully dissected along a subperiosteal plane to identify the AEA. The vessel was exposed within the orbit and dissected.

**Results:** Data on technical difficulties, complications, the learning curve and anatomical variations were gathered.

**Conclusion:** An endonasal endoscopic approach to the AEA within the orbit was shown to be feasible. Identifying the artery is not difficult, and this technique avoids external incisions. This approach appears to be an excellent alternative for approaching the AEA. Further clinical studies are needed to demonstarte the benefits of this technique.

## INTRODUCTION

Nose bleeding is a common complaint that affects from10% to 12% of the population. About 10% of these patients seek medical help.^1^ Patients and family members may feel anxious about this situation, which requires prompt medical therapy. Treatment generally consists of evaluating the patient as a whole, assessing the status of coagulation, and anterior/posterior nasal tamponade. About 1% of these patients require a surgical procedure after not responding to conservative measures.^1,^^2^

Bleeding points are better located and controlled by endoscopic surgery.^3^ Endoscopic ligature of the sphenopalatine artery is a safe and well-established technique for treating posterior epistaxis;^1,^^4^ in some cases, however, ligature of the anterior ethmoidal artery becomes necessary to control bleeding.^5,^^6^ In 1946, Weddell described a ligature procedure for the anterior ethmoidal artery by an external approach using the Lynch incision.^7^ Since then, anatomical parameters have been defined and this method has become a standard and effective approach to treat nasal bleeding from the ethmoid artery.^8^ Nevertheless, there are complications of the external approach such as scarring, edema, facial ecchymosis, and damage to the medial canthal ligament.^9^

Although endoscopy is widely accepted and used for treating several diseases of the nose and cranial base, its use for ligating the anterior ethmoidal artery is not widely disseminated. Few papers have been published to normatize the endoscopic approach to this blood vessel. The purpose of this study was to assess the technical applicability of periorbital ligation of the anterior ethmoidal artery by a transethmoidal endoscopic approach. We also evaluated the dehiscence of the anterior ethmoid canal, the distance from the anterior ethmoidal artery to the cranial base, and the learning curve.

## MATERIAL AND METHOD

The institutional review board approved this study (protocol 113/04), which consisted of dissecting 25 consecutive cadavers (50 nasal fossae) in the Death Investigation Unit at the São Paulo University (SVOC-USP). The cause of death was not taken into account. Subjects aged over 18 years only were included. The following exclusion criteria were applied:

- History of craniofacial trauma;
- History of nasosinusal surgery;
- Presence of any disease that altered the nasal anatomy (sinusitis, polyps, etc).

All procedures were documented on video.

### Dissection technique

**Figure 1:**
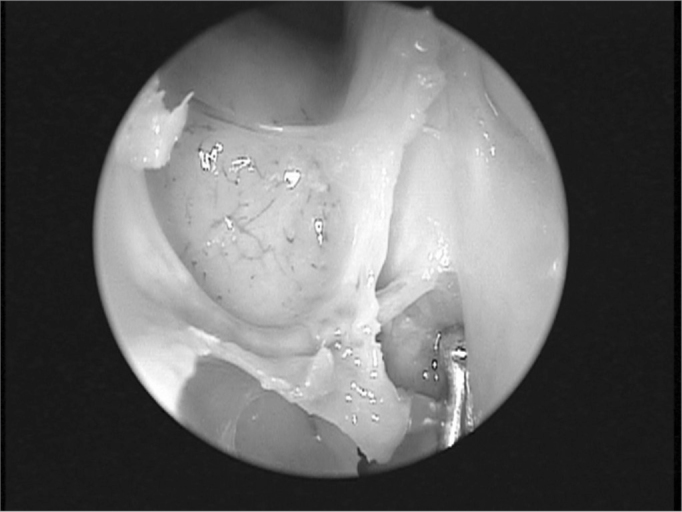
Exposed anterior ethmoid artery - subperiosteal plane, left orbit - during endoscopic dissection of a cadaver (45° endoscope). Note the dehiscent intranasal trajectory in a posterior-anterior direction.

**Figure 2:**
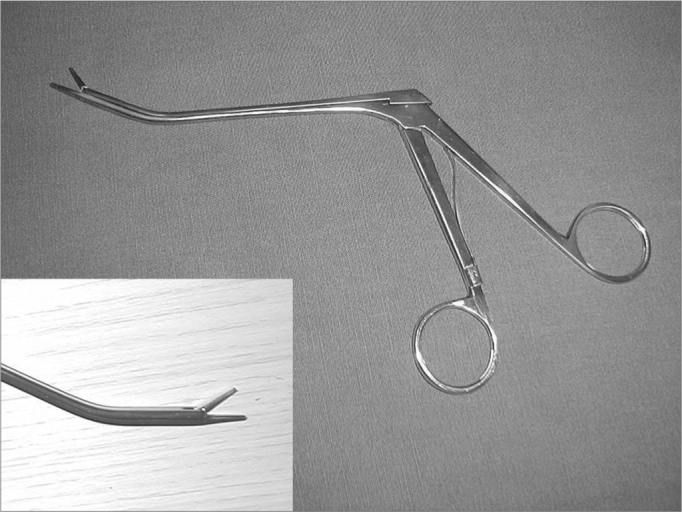
Angled forceps with Ligclip, used in procedures; detailed image of its tip.

**Figure 3:**
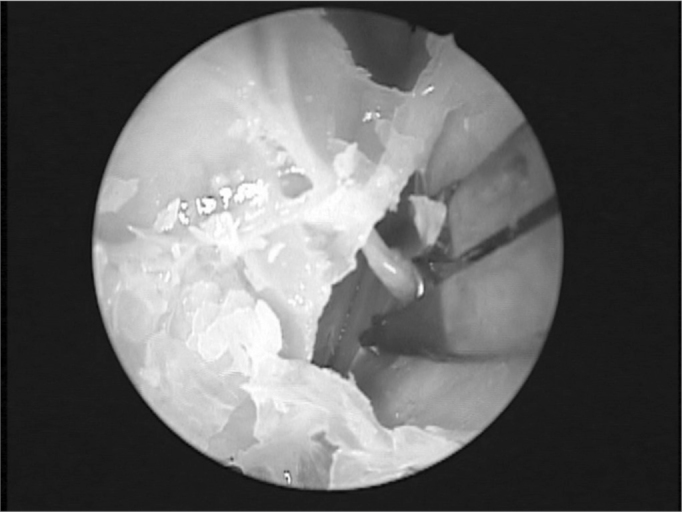
Forceps with Ligclip LT 200 around the artery on the medial wall of the orbit (45° endoscope).

**Figure 4:**
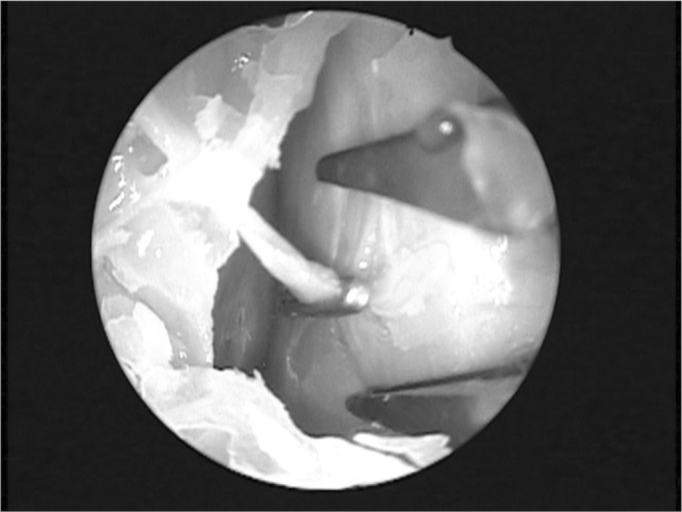
Ligclip on the left anterior ethmoid artery - subperiosteal plane.

Endonasal surgery instruments were used in the dissections (cottle, angled and straight prehension forceps, ligaclip, cutting forceps, seekers, and frontal sinus curettes), a 4 mm 0° Storz Hopkins^®^, endoscope, a 4 mm 45° Storz Hopkins^®^ endoscope, and a video-endoscopy system. The same surgeon with experience in endoscopic techniques carried out all dissections. Both nasal fossae were adequately cleaned with water and gauze before carrying out anterior right ethmoidectomy. The anterior ethmoid bone trabeculae should be fully removed to expose the lamina papyracea and the cranial base. The upper portion of the uncinate process was removed to expose adequately the frontal recess. Next, the anterior ethmoidal artery was located on the cranial base, posterior to the frontal recess.^10^ The artery was palpated with a seeker along its course on the ethmoid roof. Data was gathered about the integrity of the bony canal that surrounds the anterior ethmoidal artery to classify it as: intact canal, partially dehiscent canal, or completely dehiscent canal. The distance between the artery and the cranial base was also measured. The next step was to partially remove the lamina papyracea on the anterior region adjacent to the anterior ethmoidal artery. The periorbital area was identified and carefully dissected to indentify and expose the anterior ethmoidal artery within the orbit. In this situation the artery was identified as it entered the anterior ethmoid foramen (Fig. 1). A 30° ligaclip forceps was used to ligate the artery. The ligclip angle is important for adequately visualizing the anterior ethmoidal artery during this procedure (Fig. 2). A straight ligaclip forceps (open clip) would make it harder to visualize the artery with a 0° degree endoscope. LT200 titanium clips (Figs. 3 and 4) were used. The same procedure was repeated in the left side.

We identified and noted the inherent difficulties of this procedure, the complications, the learning curve, and the anatomical variants of the anterior ethmoidal artery, such as: the distance between the artery and the cranial base classified in three groups (<2.5mm; >2.5 and <5mm; >5mm), and the degree of dehiscence.

**Figure 5:**
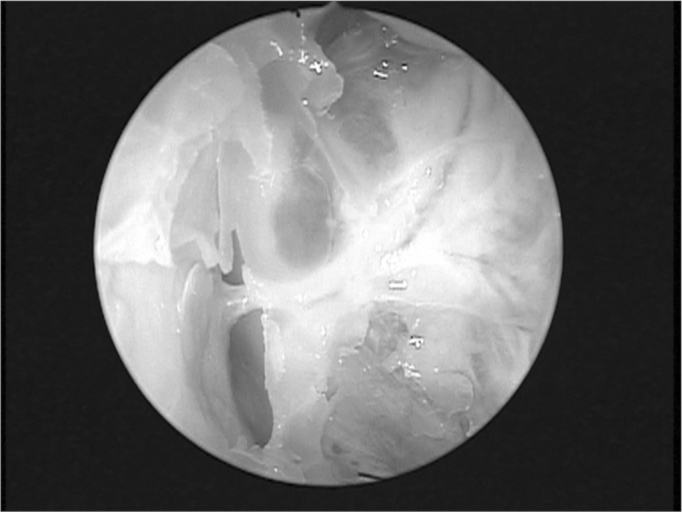
Right anterior ethmoid artery within the bony canal and exposed in the orbit after removing the lamina papyracea. Note the proximity to the cranial base.

Data were stored in a database for analysis using the SPSS 10.0 software for Windows^®^. The Mann-Whitney U test was applied to analyze measurement differences among ethnic groups and sexes. Statistically significant values were *p* ≤ 0.05.

## RESULTS

There were 10 male cadavers (40%) and 15 female cadavers (60%). Age ranged from 39 to 83 years (mean: 61 ± 13 years).

Fifty nasal fossae were evaluated. Ligature of the anterior ethmoidal artery was successful in 98.5% of cases (47 nasal fossae). Partial dehiscence of the anterior ethmoid canal was found in 40% of cases; complete dehiscence was found in 24% of cases (Table 1). The canal was intact in 36% of cases. There was no statistically significant difference in dehiscence between sexes (χ2 *p*= 0.45).

**Table 1:** Presence of dehiscence in the anterior ethmoid canal.

ETHMOID CANAL		
	Frequency	%
Complete Dehiscence	12	24,0
Partial dehiscence	20	40,0
Intact	18	36,0
Total	50	100,0

Measurements of the distance between the artery and the cranial base showed that 84% of arteries were next to the roof of the ethmoid, 4% were located from 2.5 to 5mm, and 12% were located more than 5 mm from the cranial base.

There were complications during the procedure: exposure of the orbitary fat during dissection in 8 nasal fossae (16.6%) that made it difficult to see the artery and resulted in unsuccessful ligature in 3 nasal fossae (1.5%). Most (75%) orbitary complications were found in the first 20 nasal fossae that were dissected, because of the learning curve. There were no cases of intracranial penetration and/or injury of the dura mater.

## DISCUSSION

The anterior ethmoidal artery is located in a bony canal - the anterior ethmoid canal - in its intranasal trajectory. This canal starts at the orbit in the anterior ethmoid foramen and crosses the ethmoid roof. The anterior ethmoidal artery irrigates the mucosa of the anterior ethmoid cells and the frontal sinus; it gives rise to meningeal vessels along its path in the olfactory fossa. It also irrigates the anterior third of the septum and the adjacent lateral wall of the nose,^11^ then follows along the ethmoid roof diagonally in a posterior-anterior direction (Fig. 1) to penetrate the cranium on the junction of the cribiform plate and the lateral lamella of the olfactory fossa. This is a fragile injury-prone area where cerebrospinal fluid leaks may occur.^12,^^13^ Thus, awareness of the anatomy of the anterior ethmoidal artery - an important landmark for the cranial base^14^ - is essential for managing ethmoid conditions and for safe and effective endoscopic ligature of this vessel.

Arterial blood supply to the nose comes from the internal and external carotid systems. Branches originating from the external carotid system are the sphenopalatine arteries, the greater palatine artery, and septal branches of the facial artery. The internal carotid system gives rise to the anterior and posterior ethmoidal arteries. The external carotid system dominates as the main arterial system irrigating the nose; the sphenopalatine artery is its main branch. Thus, when there is diffuse or local nasal bleeding of unidentified origin, authors recommend occlusion (ligature or cauterization) of the sphenopalatine artery.^15^

The first transnasal procedure for ligature of the sphenopalatine artery was described in 1976.^16^ During the 1990s, with the advent of endoscopy in nasal surgery, this approach became established as the treatment of choice for posterior epistaxis not responding to conservative measures.^1^ Some authors recommend occluding both the sphenopalatine and the anterior ethmoidal arteries in the same procedure in cases of severe and diffuse bleeding.^17^ Rockey et al. (2002) published a critical analysis of the surgical treatment of epistaxis. These authors found that bleeding continued in 33% of cases after ligature of the sphenopalatine artery only. Bleeding was controlled in these patients after ligature of the anterior ethmoidal artery by an external approach.^1^ A few years before, Singh and Snyderman had found that ligature of the sphenopalatine artery associated with ligature of the anterior ethmoidal artery reduced the number of recurrences, and therefore the need for additional anesthesia and surgery to control bleeding.^5,^^6^

Ligature of the anterior ethmoidal artery is classically described using the external approach. Common complications are scarring, facial edema, and damage to the medial canthal ligament, which may affect drainage in lacrimal ducts and result in epiphore. An external approach may also be used for ligating the posterior ethmoid artery; however, severe complications have been reported when using this approach for ligating this artery. Brouzas et al. reported traumatic neuropathy of the optic nerve;^18^ and Yeh et al. reported orbital apex syndrome following ligature of the posterior ethmoid artery.^17^ There probably was direct trauma to neural structures in these cases, either due to the materials that were used, or because of excessive traction on the ocular globe close to the orbital apex. The posterior ethmoid artery is very close to the optic nerve - about 5 mm distance - which precludes an adequate exposure of this vessel.

Woolford et al. (2000) carried out the first successful in vivo endoscopic endonasal ligature of the anterior ethmoidal artery. This technique is based on ligature of the ethmoidal portion of the anterior ethmoidal artery, which is usually close to the cranial base. The authors underlined the risks (intracranial and orbital) and the need for experiences surgeons.^9^ In our study we identified, exposed and ligated the anterior ethmoidal artery by dissecting along the periorbital plane. We believe that controlling the artery proximally, within the orbit, minimizes the risk of intracranial injury; the area in which the anterior ethmoidal artery penetrates the cranial base on the ethmoid roof is fragile and easy to injure. Additionally, dissecting the anterior ethmoidal artery intraorbitally avoids a complication that may occur when ligating its ethmoidal segment, namely vascular injury with retraction of the vessel into the orbit and the ensuing intraorbital hematoma, which may occur when manipulating the artery on the roof of the ethmoid.^19^ In the procedure we described, we removed part of the lamina papyracea, decompressed the orbit, and ligated the artery between the periorbital area and the medial bony wall of the orbit at the level of the anterior ethmoidal foramen.

Solares et al. (2009) published a paper on endonasal endoscopic ligature of the anterior ethmoidal artery, in which they dissected 8 cadavers (16 nasal fossae) and ligated the artery in its ethmoidal portion.^20^ There are two types of bony canal in the ethmoid roof within which the artery courses; there may be a thin bony lamina between the canal and the cranial base, which forms a type of mesentery, or there may be a thick bony layer between the canal and the cranial base. These authors realized that ligature of the anterior ethmoidal artery could not be done when the bony mesentery was absent, and therefore a thick bony layer between the canal and the roof of the ethmoid was present; this finding was present in 10 of 16 dissected nasal fossae. Ligature was effective in only three cases when a bony mesentery was present (6 nasal fossae). The authors concluded that the success rate of endonasal endoscopic ligature of the anterior ethmoidal artery on the roof of the ethmoid was 18.8%.

These numbers differ significantly from our results and from another study in which ligature of the artery was done within the orbit. Camp et al. (2009) published um study of endonasal endoscopic dissections of the anterior ethmoidal artery in 16 cadavers (32 nasal fossae).^21,^^22^ These authors described a technique that included clipping the artery within the periorbital space, as described in the present study. Camp et al. had a 100% success rate in ligating the artery, with no intracranial injury. We successfully clipped the artery in 98.5% of cases (47 nasal fossae). The three cases in which ligature of the artery was not possible were among the first nasal fossae that were dissected, where the periorbital fat was exposed; as learning progressed, this fat was no longer exposed, and ligature was possible in all remaining cases.

In relation to non-integrity of the ethmoid bony canal, artery dehiscence was present to some degree in nearly two thirds (64%) of the nasal fossae that we dissected. Moon et al.^21^ and Stammberger et al.^12^ reported lower frequencies of dehiscence (respectively 11.4% and 40%). Solares et al. found no bone dehiscence in 16 dissected canals.^20^ These results show how anatomically variable this area is. Surgeons should always be aware of the possibility of dehiscence of the bony canal that encloses the anterior ethmoidal artery; inadvertent injury of the artery can cause it to retract and an intraorbital hematoma to form.

We found that only 12% of anterior ethmoidal arteries were more than 5 mm from the ethmoid roof, which concurs with the results of Moom et al. (14.3%).^21^ Thus, the anterior ethmoidal artery is less than 5 mm from the cranial base in nearly 90% of cases. Such closeness, as well as the presence of a thick bony lamina between the anterior ethmoidal canal and the cranial base, make ligation of the anterior ethmoidal artery on the ethmoid roof difficult.

The only complication we had was exposure of the orbitary fat; we found these cases occurred early in the learning curve. Seventy-five percent of these cases occurred in the first half of cases; 25% occurred in the remaining half. The first procedures were more technically difficult - as Woolford et al. have warned - but became easier as we progressed along the learning curve. There were no intracranial lesions among our cases; this type of injury is probably associated more with manipulation and ligature of the anterior ethmoidal artery while dissecting its ethmoid segment.

## CONCLUSION

Endonasal endoscopic ligature of the anterior ethmoidal artery in the periorbital area is a feasible technique in cadaver dissections. A high success rate and few complications were found when ligature was done in the periorbital region. This technique appears to be superior - with fewer complications - compared to ligature of the ethmoid portion of the artery. Furthermore, ligature of this vessel in the periorbital area appears to present no anatomical constraints, whereas ligature of the artery on the ethmoid roof is constrained because of a higher risk of complications; these include proximity of the vessel to the cranial base and dehiscence of the ethmoid canal. Further studies are required to support the clinical applicability of endoscopic anterior ethmoidal artery ligature in the periorbital area for the treatment of epistaxis.
